# Expansion of tumor-infiltrating lymphocytes from head and neck squamous cell carcinoma to assess the potential of adoptive cell therapy

**DOI:** 10.1007/s00262-024-03691-9

**Published:** 2024-04-17

**Authors:** Sangjoon Choi, Mofazzal Hossain, Hyun Lee, Jina Baek, Hye Seon Park, Chae-Lyul Lim, DoYeon Han, Taehyun Park, Jong Hyeok Kim, Gyungyub Gong, Mi-Na Kweon, Hee Jin Lee

**Affiliations:** 1 Department of Pathology, Brain Korea 21 project, Asan Medical Center, University of Ulsan College of Medicine, 88 Olympic-ro, 43-gil, Songpa-gu, Seoul, 05505 Republic of Korea; 2 Department of Medical Science, Brain Korea 21 project, AMIST, Asan Medical Center, University of Ulsan College of Medicine, Seoul, Republic of Korea; 3 Department of Pathology, Seoul St. Mary’s Hospital, College of Medicine, The Catholic University of Korea, Seoul, Republic of Korea; 4 NeogenTC Corp, Seoul, Republic of Korea; 5 Mucosal Immunology Laboratory, Department of Convergence Medicine, Brain Korea 21 project, Asan Medical Center, University of Ulsan College of Medicine, 88 Olympic-ro 43-gil, Songpa-gu, Seoul, 05505 Republic of Korea

**Keywords:** Head and neck, Squamous cell carcinoma, Tumor-infiltrating lymphocyte, Adoptive cell transfer

## Abstract

**Background:**

Adoptive transfer of in vitro expanded tumor-infiltrating lymphocytes (TILs) has been effective in regressing several types of malignant tumors. This study assessed the yield and factors influencing the successful expansion of tumor-infiltrating lymphocytes (TILs) from head and neck squamous cell carcinoma (HNSCC), along with their immune phenotypes.

**Methods:**

TILs were expanded from 47 surgically resected HNSCC specimens and their metastasized lymph nodes. The cancer tissues were cut into small pieces (1–2 mm) and underwent initial expansion for 2 weeks. Tumor location, smoking history, stromal TIL percentage, human papillomavirus infection, and programmed death-ligand 1 score were examined for their impact on successful expansion of TILs. Expanded TILs were evaluated by flow cytometry using fluorescence-activated cell sorting. A second round of TIL expansion following the rapid expansion protocol was performed on a subset of samples with successful TIL expansion.

**Results:**

TILs were successfully expanded from 36.2% samples. Failure was due to contamination (27.6%) or insufficient expansion (36.2%). Only the stromal TIL percentage was significantly associated with successful TIL expansion (*p* = 0.032). The stromal TIL percentage also displayed a correlation with the expanded TILs per fragment (*r* = 0.341, *p* = 0.048). On flow cytometry analysis using 13 samples with successful TIL expansion, CD4 + T cell dominancy was seen in 69.2% of cases. Effector memory T cells were the major phenotype of expanded CD4 + and CD8 + T cells in all cases.

**Conclusion:**

We could expand TILs from approximately one-third of HNSCC samples. TIL expansion could be applicable in HNSCC samples with diverse clinicopathological characteristics.

**Supplementary Information:**

The online version contains supplementary material available at 10.1007/s00262-024-03691-9.

## Introduction

Head and neck squamous cell carcinoma (HNSCC) is the 6th most common cancer worldwide, and its incidence continues to rise [1]. Currently, surgery followed by radiotherapy or concurrent chemoradiotherapy is the treatment of choice for HNSCC [1]. However, the prognosis of HNSCC is still unsatisfactory because of the high incidence of tumor recurrence and/or metastasis [2]. Recurrent and/or metastatic (R/M) HNSCC generally has a poor clinical outcome and has therapeutic challenges [3, 4]. Although immune checkpoint inhibitors (ICIs) demonstrated efficacy and safety for R/M HNSCC and have recently been approved by the FDA, application of ICI therapy remains limited to a subset of tumors with programmed death-ligand 1 (PD-L1) expression [1, 4]. In the era of precision cancer therapy, a new therapeutic approach is urgently needed to provide additional treatment options and improve the clinical outcomes of R/M HNSCC patients.

An innovative strategy, known as tumor-infiltrating lymphocyte (TIL) therapy, is based on adoptive cell therapy (ACT) with the application of TILs for the treatment of cancer [5, 6]. For TIL therapy, TILs are isolated from the resected tumor specimens, expanded in culture with interleukin-2 to achieve a clinically relevant number of cells, and subsequently infused back into the patients. TIL therapy presents several advantages for treating solid tumors: (1) TILs possess diverse T-cell receptor (TCR) repertoires capable of recognizing variable tumor antigens, effectively overcoming the intratumoral heterogeneity that often leads to resistance against targeted therapy. (2) TILs, primarily composed of effector memory T cells that have been stimulated by tumor antigens in vivo, harbor chemokine receptors on their cell surfaces and thereby have better tumor-homing ability. (3) To-date, limited reports exist regarding the off-target toxicity of TIL therapy, and the process of negative selection of TCRs within TILs may contribute to the safety of this therapeutic approach.

Several trials have demonstrated the clinical benefits of TIL therapy, mainly in metastatic melanoma [7, 8], but also with other solid tumors, including breast cancer, cervical cancer, colorectal cancer, and non-small cell lung cancer [9–12]. Also, a recent study reported that TIL therapy in combination with pembrolizumab achieved a high overall response rate in R/M HNSCC [13]. The application of novel TIL therapy can address a clinical unmet need in R/M HNSCC patients who are refractory to conventional chemotherapy or immunotherapy.

Despite the promising results, the detailed process of TIL culture for preparation of the treatment and the clinicopathological characteristics associated with successful TIL culture of HNSCC specimens have not been documented previously. Here in this study, we cultured TILs from 47 specimens of primary tumors and metastatic lymph nodes (LNs) of HNSCC obtained from the oral cavity, oropharynx, and larynx of 33 patients. Next, we comprehensively investigated their clinicopathological characteristics, including stromal TIL (sTIL) percentage, human papillomavirus (HPV) infection status, and PD-L1 combined positive score (CPS), which could affect successful TIL expansion. Additionally, we analyzed the compositions of the expanded TIL using flow cytometry and performed a second round of TIL culture using a standard rapid expansion protocol (REP).

## Method

### Patient selection and data collection

**Fig. 1:**
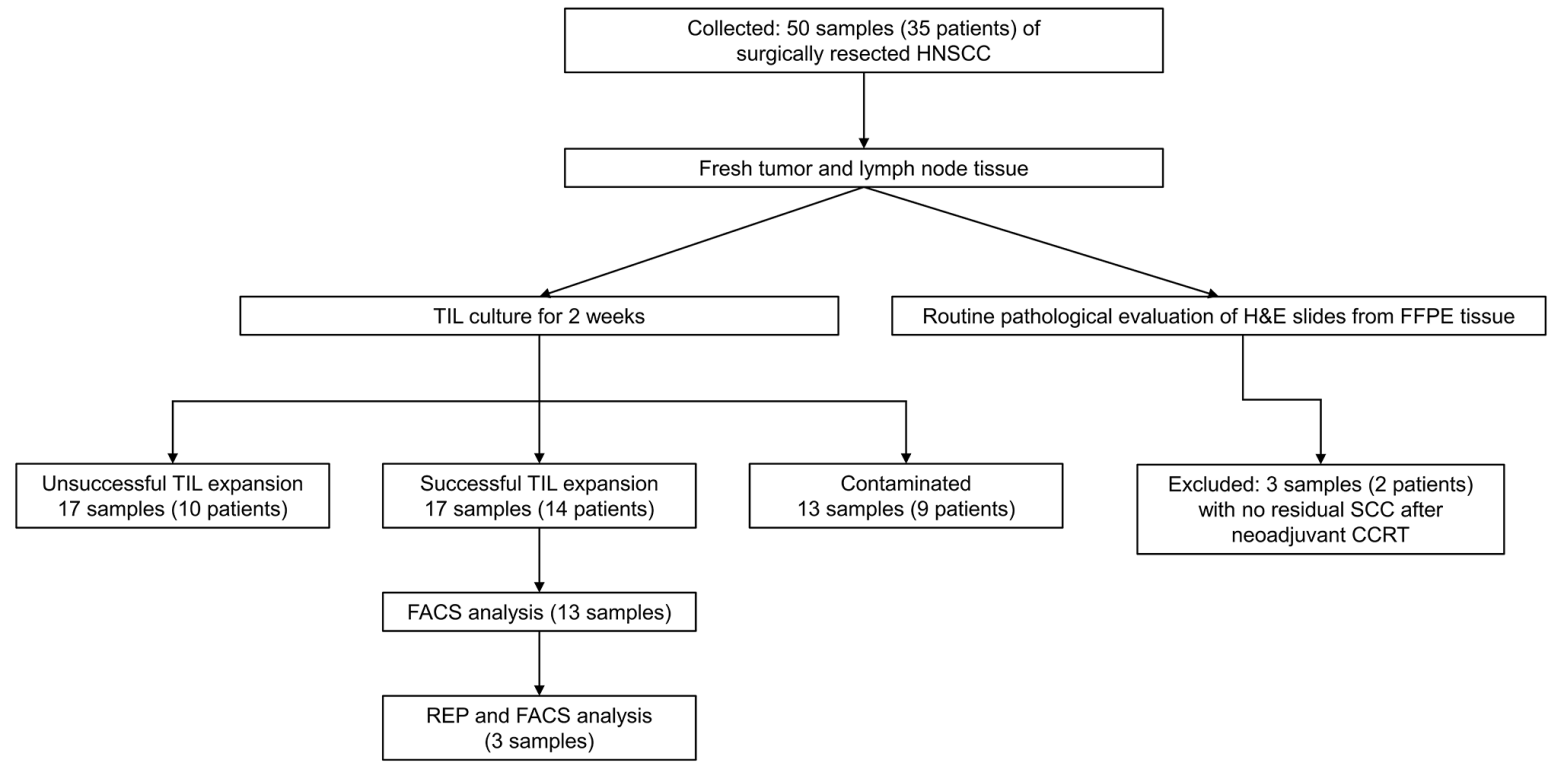
Flowchart of the sample (patient) selection and tumor-infiltrating lymphocyte expansion process. HNSCC, head and neck squamous cell carcinoma; H&E, hematoxylin and eosin; FFPE, formalin-fixed paraffin-embedded; CCRT, chemoradiation therapy; TIL, tumor-infiltrating lymphocyte; FACS, fluorescence-activated cell sorting; REP, rapid expansion protocol

Between 2020 and 2022, 35 patients were selected after obtaining their informed written consent for this single-institution study. Two patients were excluded due to no residual tumor on histopathological examination after neoadjuvant chemoradiation therapy. A total of 47 specimens from 33 patients of surgically resected HNSCC from three different anatomical locations, including tumors (*n* = 30) and their metastasized LNs (*n* = 17) were analyzed in this study. Twenty-eight samples were paired tumor and LN samples from 14 patients. One tumor sample was obtained from patient who received radiation therapy and developed local recurrence in the larynx. All remaining patients had no history of pre-operative chemotherapy or radiotherapy. We investigated the patients’ clinical information by using electronic medical records, including their age at diagnosis, sex, surgical treatments, tumor location, and smoking history. Figure 1 illustrates the flowchart of the patient selection process.

### Initial TILs expansion protocol

Within 2 hours of resection, fresh tumor and LN tissues were obtained in the Department of Pathology and brought to the laboratory (in RPMI). The presence of tumor in LNs were determined through gross examination based on the size and cut surface of the LNs. Metastatic LNs were grossly enlarged and cut surface showed well-demarcated, white-tan colored, firm nodules compared to non-metastatic lymph nodes. The cancer tissues were cut into small pieces (1–2 mm each) and placed on 24 flat bottom well plates. TIL culture media (RPMI 1640 medium; Life Technologies, Carlsbad, CA, USA) was supplemented with 10% fetal bovine serum (Corning, Corning, NY, USA), 1x ZellShield, 400 µg/ml gentamycin, 50 nM 2-mercaptoethanol (Life Technologies), and 1,000 IU/mL human recombinant interleukin-2 (Miltenyi Biotec, Bergisch Gladbach, Germany). Then, the plate was incubated at 37 ℃ in a 5% CO_2_ incubator for 14 days. Half of the medium was replaced every 2 or 3 days. After 2 weeks, the cultured TILs were counted and cryopreserved until further analysis. We set the cutoff value of successful expansion as 0.8 × 10^5^ TILs per fragment of specimen tissue [14]. Cloudy and turbid culture medium was regarded as microbiological contamination and discarded.

### Histopathologic evaluation

**Fig. 2:**
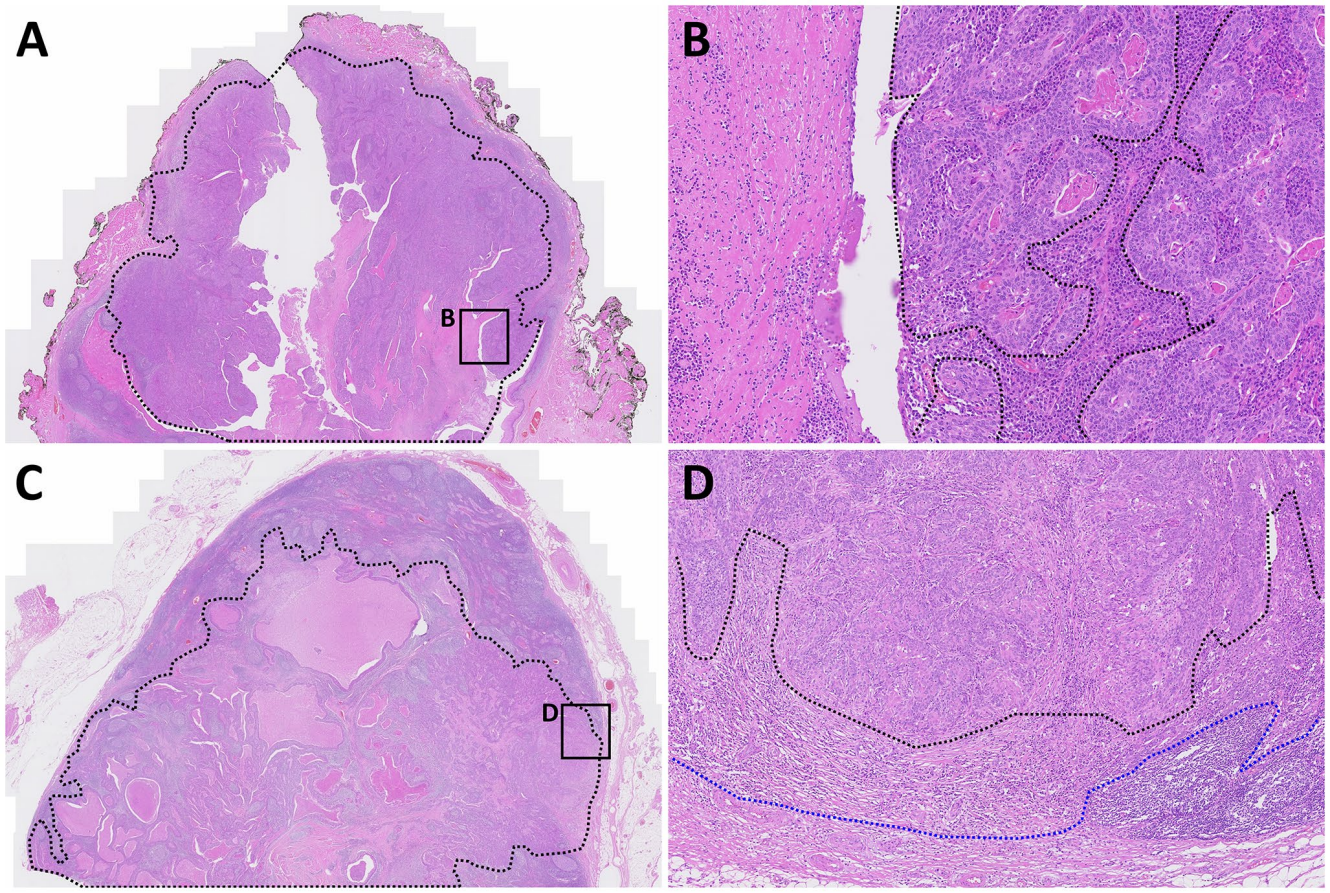
Representative hematoxylin and eosin stained images of **(A-B)** tumor [(stromal tumor-infiltrating lymphocytes (sTIL): 100%)] and **(C-D)** metastatic lymph nodes (sTIL: 80%). Black and blue dotted lines represent tumor area and desmoplastic stromal area, respectively

After tissue sampling for TIL culture, residual specimens for pathologic staging were kept for routine histological evaluation, the generation of formalin-fixed paraffin-embedded (FFPE) blocks, and hematoxylin and eosin (H&E) staining (4 μm thick sections). Pathological information, including the American Joint Committee on Cancer (AJCC) stage, tumor differentiation, largest tumor size, and the percentage of sTILs were determined by slide review. sTILs were evaluated in more than two representative H&E-stained slides per sample, and the percentage was defined as the area occupied by mononuclear inflammatory cells over the total stromal area according to the guidelines of the International Immuno-Oncology Biomarkers Working Group [15]. sTILs were evaluated not only in the tumor but also in metastatic LNs. Briefly, we defined the total tumor area by connecting the outlines of tumor nests at the periphery and assessed sTILs within the peritumoral stroma inside this area. In addition, stromal area outside the tumor outline was included in the sTIL calculation if a desmoplastic reaction was present. The same method was applied to calculate sTILs in metastatic LNs and we excluded lymphocytes belonging to normal LN structures. Representative example of H&E images of sTIL calculation in tumor and LNs are shown in Fig. 2. The most representative tumor slides were selected from each case for immunohistochemical staining of p16, PD-L1, and in situ hybridization (ISH) of HPV.

### Immunohistochemical study for p16 and PD-L1

The PD-L1 immunostaining procedure utilized a 22C3 pharmDx kit (Agilent Technologies, Carpinteria, CA, USA) with a Dako Autostainer Link 48 system (Agilent Technologies). To assess PD-L1 expression, the CPS was used. The CPS was calculated by the ratio of the number of PD-L1-stained cells (encompassing viable tumor cells, lymphocytes, and macrophages) to the total number of viable tumor cells, as described previously [16].

A primary antibody against p16INK4a (1:6, clone E6H4, mouse mAb, Ventana Medical Systems, Oro Valley, AZ, USA) was used for immunostaining. The p16 immunostaining results were interpreted as positive when both the tumor nucleus and cytoplasm exhibited moderate or strong immunoreactivity in > 75% of the tumor cells [17].

### In situ hybridization

HPV infection status was determined using DNA ISH with the INFORM HPV III Family 16 Probe (B) from Ventana Medical Systems. This probe specifically identifies high-risk HPV subtypes including 16, 18, 31, 33, 35, 45, 52, 56, 58, and 66. A positive ISH result was assigned when nuclear staining was evident in > 70% of the tumor cells [17].

### Rapid expansion protocol

In the REP step, the TILs were cultured with irradiated (50 Gy) allogeneic peripheral blood mononuclear cells from one healthy donor in REP medium (AIM-V medium; Life Technologies) supplemented with 3% Serum, 2,000 IU/mL human recombinant interleukin-2, and 30 ng/mL human anti-CD3 antibody (clone OKT3; Miltenyi Biotec) in a T-Flask (Wuxi NEST Biotechnology, Jiangsu, China) or G-Rex (Wilson Wolf, Saint Paul, MN, USA) with media replacement every 2 or 3 days. After 14 days, the post-REP TILs were collected and cryopreserved until further analysis.

### Immunophenotyping of TILs

The characteristics of the expanded cells were evaluated by fluorescence-activated cell sorting (FACS). The assessment was carried out by FACS (BD-Lyric, San Diego, CA, USA) using different antibodies including CD3-APC-Cy7 (Biolegend, San Diego, CA, USA), CD4-FITC (BD, Franklin Lakes, NJ, USA), CD8-Percp-Cy5.5 (BioLegend), CD45RA-PE (BioLegend), CCR7-PE-cy7 (BioLegend), and CD45-BV510 (BioLegend). Compensation was done by using Flowjo_v10.80 software (Tree Star, Ashland, OR, USA). The following gating strategy was used: forward versus side scatter gating, CD45 + and CD3+, CD4 + and CD8+, and CCR7 and CD45RA. CD4 + and CD8 + T cells are subclassified as effector memory T cells (T_em_, CD45RA-CCR7-), effector T cells (T_eff_, CD45RA + CCR7-), central memory T cells (T_cm_, CD45RA-CCR7+), or naïve T cells (T_naïve_, CD45RA + CCR7+) [18].

### Statistical analysis

Statistical analyses and visualization were performed using GraphPad Prism software version 9.4.1 and R (https://cran.r-project.org). The correlations between clinicopathological parameters and TIL expansion status were analyzed using Fisher’s exact test and the chi-square test. TILs per fragments and total cultured TILs after initial expansion were compared using Mann Whitney-U tests and Kruskal-Wallis tests. *P* values under 0.05 were considered statistically significant. Spearman correlation analysis was performed to assess the relationships between sTIL percentage, PD-L1 CPS, TILs per fragment, and total cultured TILs.

## Results

### Clinicopathological characteristics of the patients

Table 1 summarizes the baseline clinicopathological characteristics of 33 patients with HNSCC. The mean age of the patients was 61 years, and 78.8% were male. Samples were obtained from primary tumors (*n* = 16), metastatic LNs (*n* = 3), and both primary tumor and LNs (*n* = 14). The most common primary tumor location was the oral cavity (*n* = 20), followed by the oropharynx (*n* = 11) and the larynx (*n* = 2). Eighteen patients (54.5%) had a smoking history. The average tumor size was 3.6 ± 1.5 cm (mean ± standard deviation [SD]). p16 IHC and HPV ISH were performed for 17 and 13 patients, respectively, and 10 (58.8%) and 9 (69.2%) patients tested positive. PD-L1 IHC was performed for 17 patients and all exhibited PD-L1 positivity (CPS ≥ 1) with an average CPS of 39.8 ± 35.0 (mean ± SD). The average sTIL percentage was 48.2 ± 33.0 (mean ± SD).

**Table 1:** Baseline characteristics of 33 patients

		Total
		*N* = 33
Sex		
Male		26 (78.8)
Female		7 (21.2)
Age (mean)		61.0 ± 13.8
Samples		
Tumor		16 (48.5)
Metastatic LN		3 (9.1)
Both tumor and LN		14 (42.4)
Tumor type		
Primary		32 (97.0%)
Recurrent		1 (3.0%)
Differentiation		
WD		6 (18.2)
MD		26 (78.8)
PD		1 (3.0)
Location		
Oral cavity		20 (60.6)
Oropharynx		11 (33.3)
Larynx		2 (6.1)
Smoking history		
(-)		15 (45.5)
(+)		18 (54.5)
Smoking duration (pack-years)		
0		15 (45.5)
1–20		9 (27.3)
21–40		4 (12.1)
≥ 41		5 (15.1)
Tumor size (mean, cm)		3.6 ± 1.5
p16 immunoreactivity (*n* = 17)		
(-)		7 (41.2)
(+)		10 (58.8)
HPV infection (*n* = 13)		
(-)		4 (30.8)
(+)		9 (69.2)
Stromal TIL (%)		48.2 ± 33.0
PD-L1 CPS (22C3) (*n* = 17)		39.8 ± 35.0
Postoperative therapy		
CCRT		10 (21.3)
RT		19 (40.4)
None		4 (8.5)

LN, lymph node; WD, well differentiated; MD, moderately differentiated; PD, poorly differentiated; HPV, human papilloma virus; TIL, tumor-infiltrating lymphocyte; PD-L1, programmed death-ligand 1; CPS, combined positive score; CCRT, concurrent chemoradiation therapy; RT, radiation therapy

### The yield of TIL culture and associated clinicopathological characteristics

The initial expansion of TILs was successful for 17 of 47 (36.2%) specimens. The average TILs per fragment and the total cultured TILs were 5.5 ± 4.3 (x 10^5^, mean ± SD) and 36.6 ± 33.6 (x 10^6^, mean ± SD), respectively. A low cell growth rate (TILs less than the cutoff value (0.8 × 10^5^ per fragment) was observed in 17 (36.2%) samples. The remaining 13 (27.6%) specimens were discarded due to contamination at various stages of the expansion process.

The associations between successful TIL expansion and clinicopathological parameters are shown in Table 2. Higher sTILs (*p* = 0.032) and moderate-to-poorly differentiated tumors (*p* = 0.014) were significantly associated with successful TIL expansion. However, no significant difference was observed between sTIL and tumor differentiation (Supplementary Fig. 1). Also, the successful TIL expanded group displayed a tendency for higher PD-L1 CPS, although the difference did not reach statistical significance (*p* = 0.094). Sample type, tumor size, AJCC stage, tumor location, smoking history or duration, and p16 and HPV status were not related to successful TIL expansion.

**Table 2:** Clinicopathologic characteristics of 34 samples by successful and unsuccessful tumor-infiltrating lymphocyte (TIL) expansion groups

	Insufficient TIL expansion	Successful TIL expansion	
	*N* = 17	*N* = 17	*p* value
TILs per fragment (x10^5^, mean ± SD)	0.1 ± 0.2	5.5 ± 4.3	< 0.001
Total cultured TILs (x10^6^, mean ± SD)	0.4 ± 0.7	36.6 ± 33.6	< 0.001
Age	58.9 ± 16.2	62.9 ± 13.3	0.445
Sex			
Male	4 (23.5)	7 (41.2)	0.463
Female	13 (76.5)	10 (58.8)	
Sample type			
LN	6 (35.3)	9 (52.9)	0.490
Tumor	11 (64.7)	8 (47.1)	
Tumor size	4.1 ± 1.9	3.4 ± 1.1	0.305
AJCC T stage			
1	1 (5.9)	1 (5.9)	0.941
2	4 (23.5)	6 (35.3)	
3	8 (47.1)	6 (35.3)	
4	4 (23.5)	4 (23.5)	
AJCC N stage			
0	2 (11.8)	2 (11.8)	0.433
1	6 (35.3)	8 (47.1)	
2	8 (47.1)	4 (23.5)	
3	1 (5.9)	3 (17.6)	
Histology			
WD	6 (35.3)	0 (0.0)	0.014
MD	11 (64.7)	16 (94.1)	
PD	0 (0.0)	1 (5.9)	
Location group			
Oral cavity	1 (5.9)	1 (5.9)	0.730
Oropharynx	11 (64.7)	8 (47.1)	
Larynx	5 (29.4)	8 (47.1)	
Smoking history			
(-)	6 (35.3)	8 (47.1)	0.727
(+)	11 (64.7)	9 (52.9)	
Smoking duration (pack-years)			
0	6 (35.3)	8 (47.1)	0.633
1–20	4 (23.5)	5 (29.4)	
21–40	4 (23.5)	1 (5.9)	
≥ 41	3 (17.6)	3 (17.6)	
p16 immunoreactivity			
(-)	3 (42.9)	4 (33.3)	1.000
(+)	4 (57.1)	8 (66.7)	
HPV ISH			
(-)	2 (33.3)	3 (30.0)	1.000
(+)	4 (66.7)	7 (70.0)	
Stromal TIL (%, mean ± SD)	40.3 ± 27.8	63.2 ± 30.8	0.032
PD-L1 CPS (22C3)	25.2 ± 25.8	63.0 ± 37.7	0.094

SD, standard deviation; LN, lymph node; AJCC, American Joint Committee on Cancer; WD, well differentiated; MD, moderately differentiated; PD, poorly differentiated; HPV, human papilloma virus; ISH, in-situ hybridization; TIL, tumor-infiltrating lymphocyte; PD-L1, programmed death-ligand 1; CPS, combined positive score

### Clinicopathological characteristics based on HPV status and specimen type

In subgroup analysis based on HPV ISH status, early AJCC T and N stages, oropharynx origin, and positive p16 expression were significantly associated with HPV infection (Supplementary Table 1). When analysing subgroups according to the type of specimen (tumor and LN), we observed no significant difference as shown in Supplementary Table 2.

### TIL expansion ratio based on clinicopathological characteristics

**Fig. 3:**
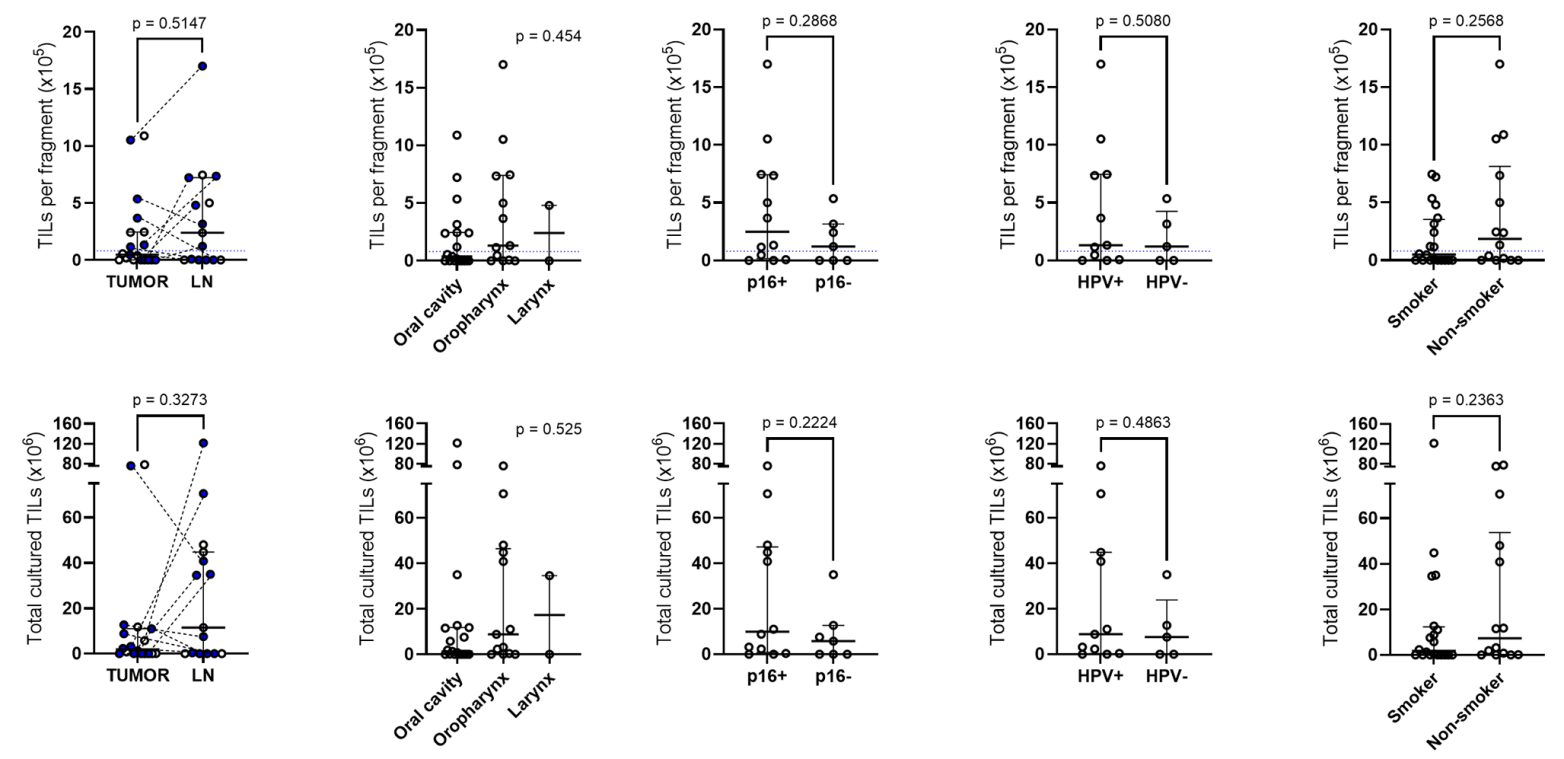
The difference of tumor-infiltrating lymphocytes (TILs) per fragment (upper panel) and total cultured TILs (lower panel) between sample types (tumor and lymph node), location, p16 positivity, human papilloma virus infection, and smoking history. Blue dots represent paired samples from the same patients. Blue dotted line represents the cutoff (0.8 × 10^5^ cells per fragment) of successful TIL expansion

**Fig. 4:**
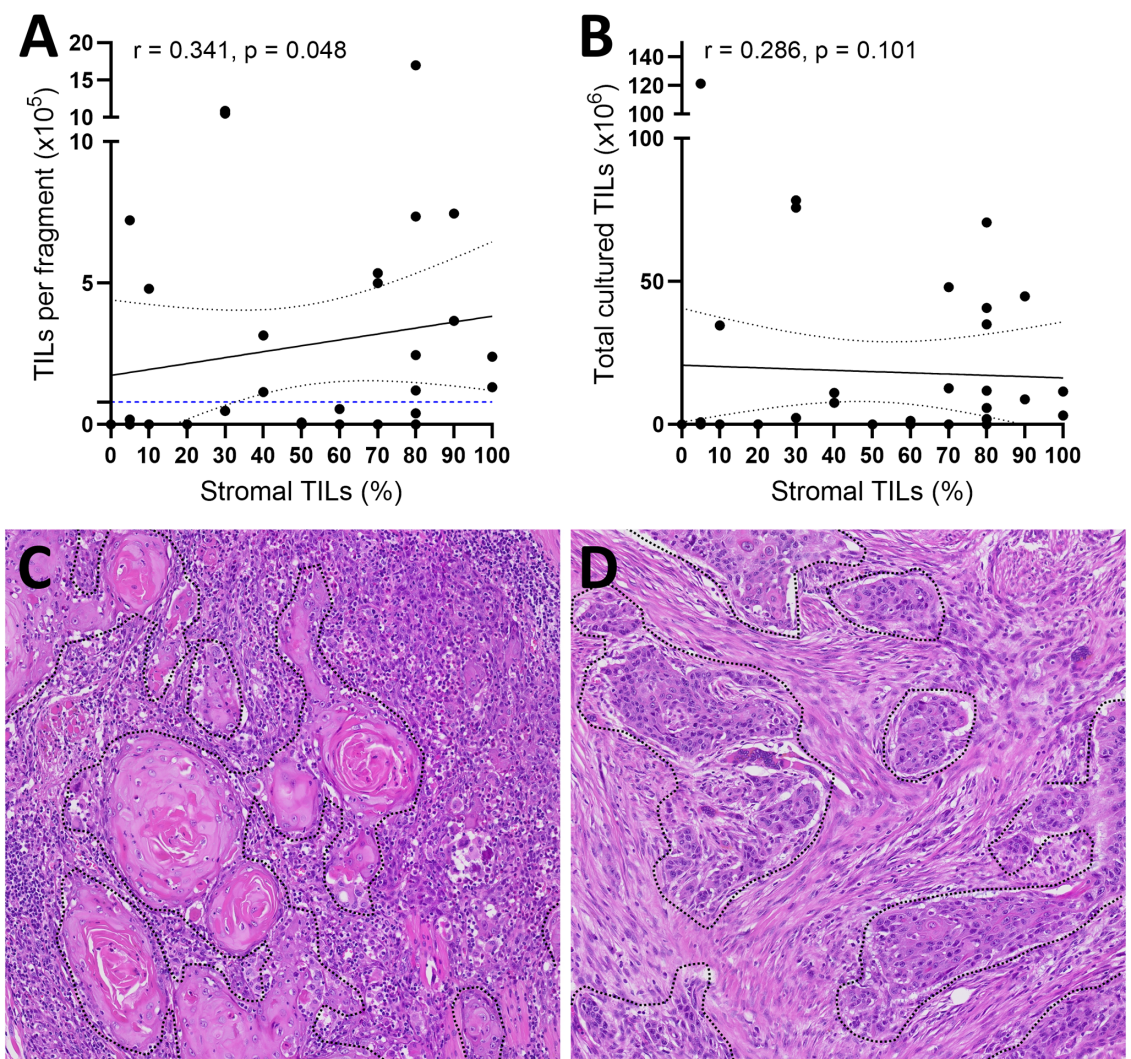
Relationship between (**A**) stromal tumor-infiltrating lymphocytes (sTIL) and expanded TILs per fragment; (**B**) sTIL and total cultured TILs; Representative hematoxylin and eosin stained slides of (**C**) of high sTIL (100%) and (**D**) low sTIL (5%). Black dotted lines in (C) and (D) represent tumor area

Expanded TILs per fragment and cultured total TILs showed no statistically significant differences among various clinicopathological parameters, including sample types, anatomical location of the primary tumors, p16-positivity, HPV infection status, and smoking history (Fig. 3). Stromal TILs evaluated on H&E slides showed a weak but significant correlation with the expanded TILs per fragment (*r* = 0.341; *p* = 0.048), but did not show a significant correlation with total cultured TILs (*r* = 0.286; *p* = 0.101)(Fig. 4A-B). When restricted to tumor samples (*n* = 19), sTILs and TILs per fragment showed similar correlation (*r* = 0.434; *p* = 0.064) but did not achieve statistical significance. sTILs and total TILs did not show correlation (*r* = 0.368; *p* = 0.121). LN samples (*n* = 15) displayed no significant association between sTIL and expanded TIL numbers (Supplementary Fig. 2). PD-L1 CPS was not significantly correlated with TILs per fragment (*r* = -0.083; *p* = 0.701) or total culture TILs (*r* = -0.126; *p* = 0.558). Representative H&E images of HNSCC with high and low sTIL are shown in Fig. 4C-D.

### Immunophenotype of expanded TILs

We evaluated the phenotypes of the successfully expanded TILs from 13 of 17 samples by FACS and the subsequent REP in 3 of 13 samples. The clinicopathological parameters and TIL phenotypes after the initial two weeks of expansion are summarized in Table 3. and the post-REP results of 3 cases are shown in Table 4.

**Table 3:** Results of flow cytometry in 13 samples (from 12 patients) with successful tumor-infiltrating lymphocyte expansion

Pt no	Location	Sample	p16	HPV	PD-L1 CPS	sTIL (%)	TILs/ fragment (x10^5^)	Total TIL (x10^6^)	Lympho cyte (%)	T cell (%)	CD8 + T cell (%)	CD8 + T_cm_ (%)	CD8 + T_naïve_ (%)	CD8 + T_em_ (%)	CD8 + T_eff_ (%)	CD4 + T cell (%)	CD4 + T_cm_ (%)	CD4 + T_naïve_ (%)	CD4 + T_em_ (%)	CD4 + T_eff_ (%)
1	Oropharynx	Tumor	+	+	NA	90	3.67	8.8	77.30	42.70	39.90	4.32	0.50	85.70	9.50	51.80	7.04	2.53	88.90	1.51
2	Oropharynx	Tumor	+	+	90	30	10.52	75.8	69.58	71.67	17.73	0.45	0.00	93.67	5.88	75.06	0.85	0.05	96.85	2.24
	Oropharynx	LN	+	+	NA	80	17.00	40.8	63.98	66.93	11.79	0.20	0.00	89.70	10.10	82.53	0.37	0.06	96.10	3.48
3	Oropharynx	LN	+	+	NA	90	7.46	44.8	70.23	54.71	40.24	0.32	0.06	83.31	16.30	47.92	0.71	0.65	92.56	6.08
4	Oropharynx	LN	+	NA	NA	70	5.00	48.0	49.40	90.70	43.00	2.70	1.92	75.00	20.30	37.90	4.11	4.11	86.20	5.58
5	Oropharynx	LN	+	+	NA	80	7.35	70.6	42.40	52.90	56.40	1.50	0.63	85.00	12.80	18.30	1.46	2.93	73.20	22.40
6	Oral cavity	Tumor	NA	NA	NA	30	10.89	78.4	43.20	11.50	8.97	1.18	1.18	89.60	7.99	79.70	0.40	0.13	99.00	0.50
7	Oral cavity	Tumor	NA	NA	NA	80	2.46	11.80	85.20	95.10	49.10	0.00	0.00	98.10	1.89	50.40	0.12	0.00	98.80	1.05
8	Oral cavity	LN	NA	NA	25	5	7.22	121.4	79.44	89.61	6.45	0.65	0.65	75.16	23.53	89.02	0.41	0.08	95.90	3.61
9	Oral cavity	LN	+	+	NA	80	3.15	7.56	60.40	80.00	44.00	10.40	6.71	55.20	27.70	37.00	16.10	6.16	74.20	3.53
10	Oral cavity	LN	-	-	NA	80	1.21	35.0	79.40	80.50	9.22	20.80	24.00	36.40	18.90	80.90	17.80	3.97	73.60	4.54
11	Oral cavity	LN	NA	NA	NA	100	2.40	11.5	93.20	92.00	62.00	3.25	0.51	87.60	8.62	33.80	12.60	2.06	81.20	4.21
12	Larynx	LN	NA	NA	NA	10	4.80	34.6	54.00	92.20	7.49	1.61	1.61	89.50	7.24	89.60	1.84	0.99	94.90	2.31

Pt, patient; HPV, human papilloma virus; PD-L1, programmed death-ligand 1; sTIL, stromal tumor-infiltrating lymphocyte; cm, central memory; eff, effector; em, effector memory; LN, lymph node; NA, not available

**Table 4:** Results of flow cytometry in 3 samples after the rapid expansion protocol

Pt no	Sample	Location	p16	HPV	PD-L1	sTIL (%)	TILs/ fragment (x10^5^)	Total TIL (x10^6^)	Lymphocyte (%)	T cell (%)	CD8 + T cell (%)	CD8 + T_cm_ (%)	CD8 + T_naïve_ (%)	CD8 + T_em_ (%)	CD8 + T_eff_ (%)	CD4 + T cell (%)	CD4 + T_cm_ (%)	CD4 + T_naïve_ (%)	CD4 + T_em_ (%)	CD8 + T_eff_ (%)	fold change	Culture flask
2	Tumor	oropharynx	+	+	90	30	10.52	75.84	68.13	72.02	18.81	0.33	0.00	92.52	7.15	74.69	0.38	0.00	98.28	1.34	2530	T-flask
7	Tumor	oral cavity	NA	NA	NA	80	2.46	11.80	90.50	82.20	30.30	0.08	0.08	99.60	0.22	66.30	0.22	0.02	99.00	0.73	960	G-rex
8	LN	oral cavity	NA	NA	25	5	7.22	121.44	88.20	98.20	21.20	0.68	0.83	19.30	79.20	76.50	0.90	0.95	61.00	37.20	1635	G-rex

HPV, human papilloma virus; PD-L1, programmed death-ligand 1; sTIL, stromal tumor-infiltrating lymphocyte; cm, central memory; eff, effector; em, effector memory; LN, lymph node, NA; not available

**Fig. 5:**
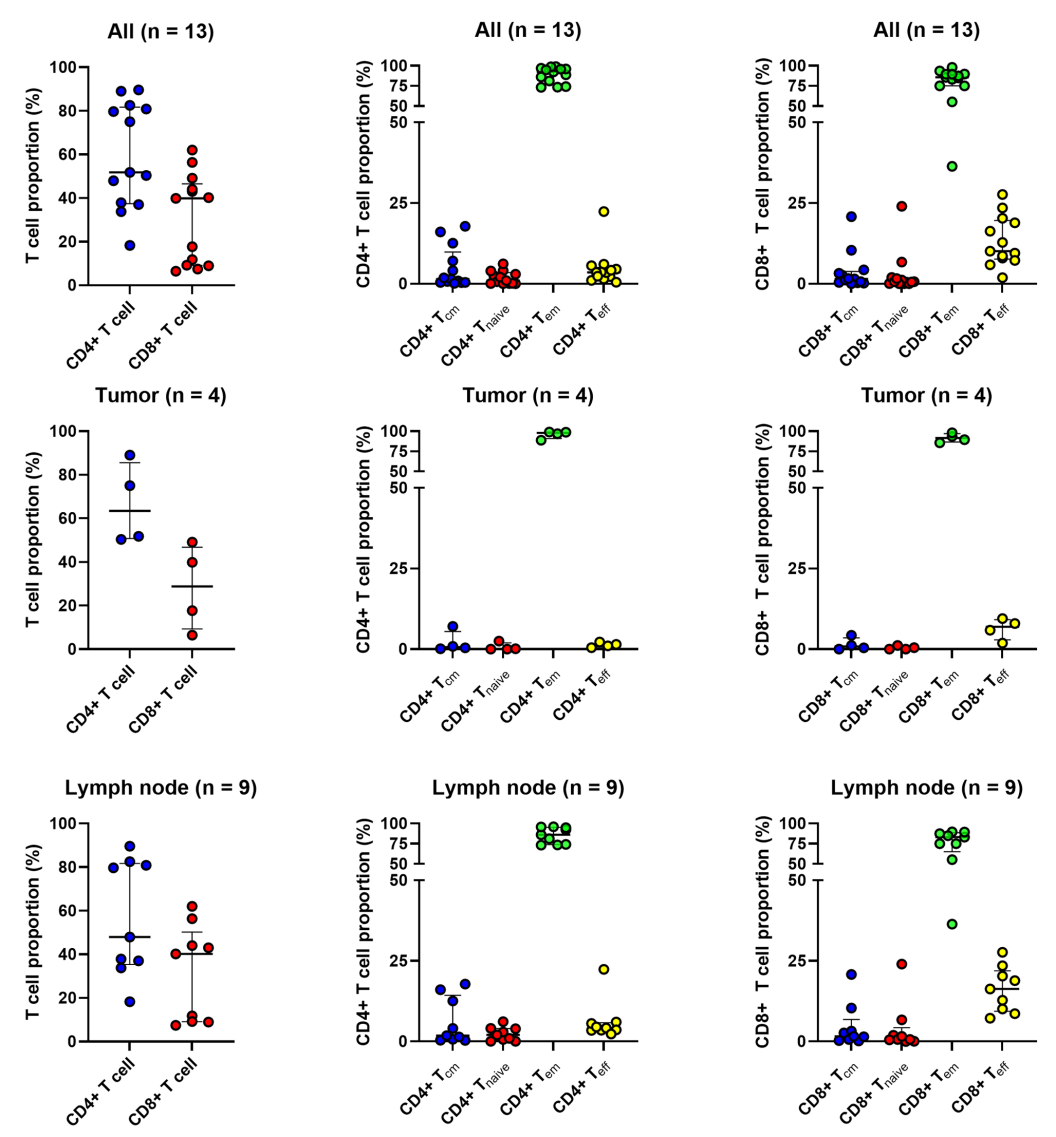
Immunophenotypes of expanded tumor-infiltrating lymphocytes from all samples (*n* = 13) (upper panel), tumor (*n* = 4) samples (mid panel), and lymph node (*n* = 9) samples with successful expansion

T cell proportions and subset populations of expanded TILs are summarized in Fig. 5. Following the initial expansion, TILs exhibited a predominance of CD4 + T cells in 9 cases (69.2%) and CD8 + T cells in 4 cases (30.7%). In all 13 cases, the major subset of T cells was T_em_, whereas the proportion of T_cm_ and T_naïve_ were notably low. The median proportions of CD4 + T cell subsets—T_em_, T_eff_, T_cm_, & T_naïve_—within the samples were 92.6%, 3.3%, 1.5%, and 1.0%, respectively. Similarly, the median proportion of CD8 + T cell subsets—T_em_, T_eff_, T_cm_, & T_naïve_—were 85.7%, 10.1%, 1.5%, and 0.6%, respectively. A predominance of CD4 + T cells along with higher proportion of CD4 + T_em_ and CD8 + T_em_ were consistently identified when classified into sample types (tumor and LNs)(Fig. 5.) and HPV infection status (HPV-positive and HPV-negative/unknown) (Supplementary Fig. 2).

**Fig. 6:**
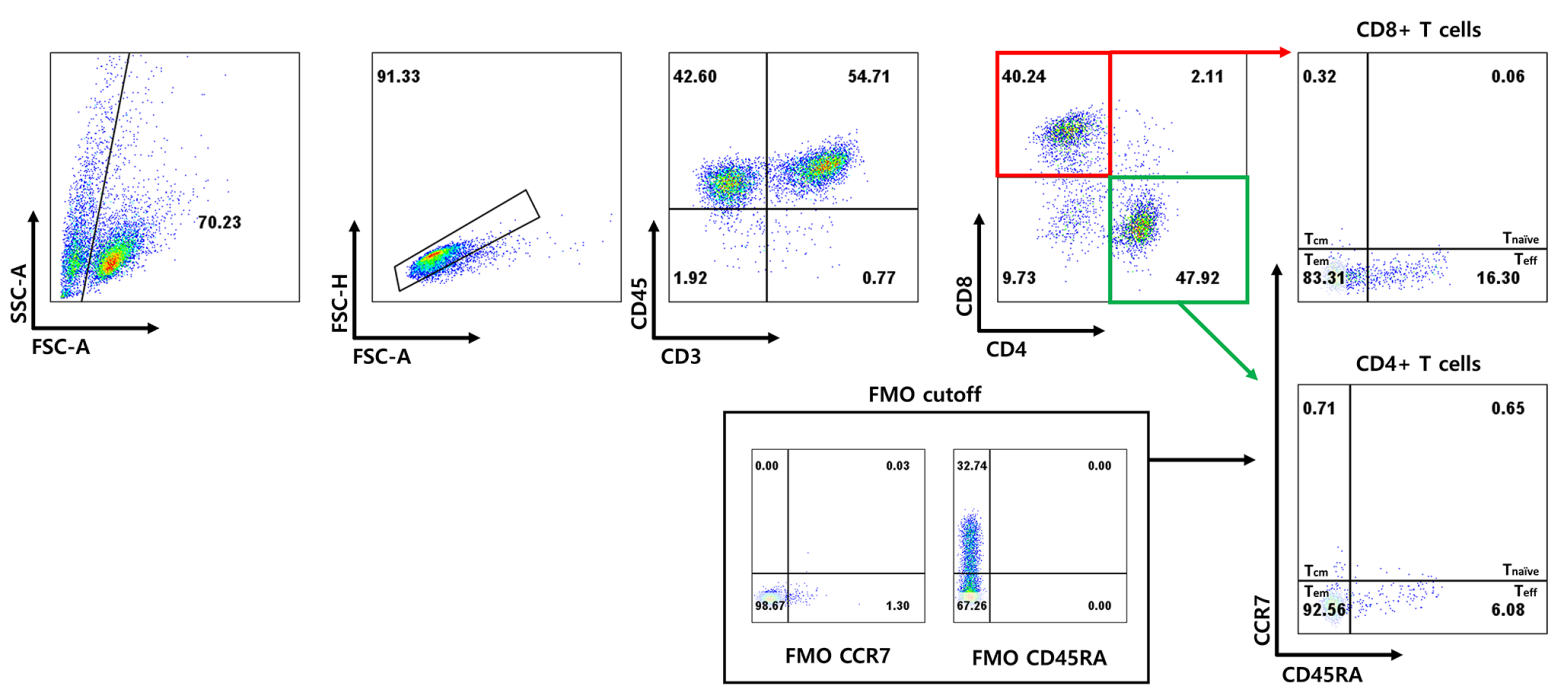
Representative result of expanded tumor-infiltrating lymphocytes with its T cell phenotype (patient 3)

Representative images of the FACS analysis (patient 3) are shown in Fig. 6. Among the three cases with REP, the TIL composition remained similar to that after the initial expansion, predominantly consisting of CD4 + T cells with the highest proportion of T_em_. In patient 8, the CD8 + T cell subset shifted from an initially dominant CD8 + T_em_ proportion to a higher proportion of the CD8 + T_eff_ subset after REP. The mean value of fold change was 1708 (range 960–2530). Fold change displayed a positive correlation with a higher TILs/fragment of the samples.

## Discussion

In this study, we demonstrated that TILs can be obtained from HNSCC with varying clinicopathological features. The sTIL percentage was found to be significantly correlated with successful TIL expansion, and could serve as a useful indicator for estimating the yield of initial TIL culture. Additionally, our findings showed that after initial expansion, TILs are mostly composed of CD4 + T cells with a predominant effector memory cell subset.

HNSCC is a histologically, molecularly, and immunologically heterogeneous disease [1]. Its pathophysiology varies according to its location, and its prognosis differs based on HPV infection status, sTIL percentage, and PD-L1 expression status [19–21]. Furthermore, sTIL and PD-L1 expression are recognized as predictive factors of immune checkpoint inhibitor responses [22]. We analyzed whether these prognostic and predictive clinicopathological parameters affect TIL expansion, but found that only sTIL percentage was a factor associated with successful TIL culture. Additionally, HPV infection, tumor location, sample type, and smoking history had no significant influence on the expanded TIL numbers. These findings suggest that TIL culture from HNSCC samples is not confined to specific subtypes and is feasible across a diverse spectrum of HNSCC. This also indicates the potential applicability of TIL therapy to HNSCCs with different biologic behaviors.

Few studies have described successful TIL expansion with delineation of their proportions from real world HNSCC samples. Zenga et al. investigated the functionality and tumor specificity of TILs obtained from surgically resected 31 HPV-negative HNSCCs [23]. TIL culture was successful in 77% of patients, with the expanded tumor-specific T cells demonstrating anti-tumor effect in co-cultures with patient-matched malignant cells. The density of T cell subset revealed a predominance of CD4 + T cells, which was in line with our study. Knochelmann et al. conducted TIL expansion using 9 oral cavity squamous cell carcinomas [24]. The expanded TILs exhibited varying populations of CD4 + and CD8 + T cells, depending on the individual patient, and demonstrated heterogeneous expression of inhibitory receptors.

We set the cutoff of essential TIL number per fragment after initial culture to be 0.8 × 10^5^ cells based on our previous study using breast cancer samples [18]. Excluding the contaminated 13 samples, the success rate of sufficient TIL expansion was 50% (17/34) of the samples. Although the success rate is slightly lower than previous reports of 60–91% across various cancer types [25–28], our findings indicate that an adequate number of TILs can be obtained from HNSCC samples.

We found that microbial contamination is a notable issue for TIL culture of HNSCC samples. Thirteen of 47 (27.5%) samples had to be discarded due to contamination during initial TIL culture. It is known that a higher frequency of microbiological contamination is present in head and neck cancer samples compared to other organs [29]. Antibiotics, such as penicillin, streptomycin, and gentamicin, have been utilized to prevent microbe contamination in head and neck cancer cell and organoid cultures [29–31]. Similarly, TIL culture methods incorporate antimicrobial agents in its protocol in order to limit the risk of contamination [32]. In our study, we added gentamicin to the media, but failed to prevent contamination in about one-fourth of the samples. Establishing a prophylactic or timely application of suitable antimicrobial agents is essential to enhance the success rate of TIL culture of HNSCC samples in future studies.

In our study, we observed that approximately 70% of cases had CD4 + T cells as a major lymphocyte subset after initial TIL expansions. Predominant CD4 + T cells in the TILs was reported for breast cancer (76%), with lower levels in gastrointestinal cancers (54%) and malignant melanoma (21%) [33–36]. While CD8 + T cells are traditionally recognized as key anti-cancer effectors, the use of ACT of CD4 + TILs showed a remarkable regression of tumor burden in patients with metastatic tumors [33, 35–37]. The elevated prevalence of CD4 + TILs in our study suggests that MHC-class II restricted neoantigens and reactive T cells are frequent in HNSCC. This finding also underscores the significant therapeutic potential of CD4 + TILs in ACT for HNSCC patients. We also noted that T_em_ had the highest proportion among the T cell subtypes in all cases. Considering the significance of effector memory cells in enhancing the therapeutic impact of ACT [38–40], a high proportion of the T_em_ phenotype from expanded TILs from HNSCC tissue indicates its adequacy for successful TIL therapy.

Post-REP TILs were evaluated in 3 available cases, and the proportion of T cell subsets were similar after REP. One case showed noticeable shift in the CD8 + T cell subset from T_em_ to T_eff_. The expected fold change following REP is over 1,000 since at least 1.0 × 10^10^ cells per patient are known to be essential for use in ACT [41, 42]. Although we were able to examine post-REP TILs and fold change in only three cases, two cases using G-Rex exceeded the fold change of 1,000 and one case using T-flask obtained slightly lower (960) results than the cutoff. Our findings suggest that sufficient number of TILs could be obtained and this indicates the potential viability of TIL therapy applications in HNSCC.

Our study has several limitations. First, the efficacy of the expanded TILs was not demonstrated through in vitro experiments or in vivo xenograft mouse models. The validation of the tumor reactivity of autologous TILs, and assessments of the long-term effects of expanded TILs, including in vitro phenotypic changes and in vivo TIL persistence after TIL infusion, requires further evaluation in future studies. Second, the REP process was available in a subset of cases after the initial two weeks of TIL expansion, preventing the observation of adequate fold changes in all cases. Although a shift in the CD8 + T cell subset was observed in one case, inferring the frequency of T cell subset changes and identifying the factors leading to this shift was difficult, given that not all cases underwent REP. Thirdly, p16, PD-L1 IHC and HPV ISH studies were only performed in a subset of cases, leading to an incomplete correlation analysis between these variables and successful TIL expansion. Despite these limitations, our study represents the comprehensive analysis of clinicopathological features associated with successful TIL expansion, conducted with large numbers of HNSCC tissue samples. Furthermore, we presented the detailed TIL phenotype following initial expansion and the subsequent REP processes, confirming that the expanded TILs are suitable for ACT, both quantitatively and qualitatively. We anticipate that our comprehensive study will contribute to expanding the current knowledge about TIL culture of HNSCC, and ultimately provide valuable insights for future research regarding TIL therapies.

In summary, we conducted TIL culture using HNSCC samples and analyzed their clinicopathological features. Despite antibiotic usage, HNSCC has an inherent susceptibility to contamination. Higher sTIL levels correlate with increased success rates and quantities of cultured TILs. The composition of the expanded TILs is predominantly CD4 + T cells with an effector memory phenotype.

### Electronic supplementary material

Below is the link to the electronic supplementary material.

Supplementary Material 1

## Data Availability

No datasets were generated or analysed during the current study.

## Abbreviations

ACT: Adoptive cell therapy
AJCC: the American Joint Committee on Cancer
CPS: combined positive score
FACS: fluorescence-activated cell sorting
FFPE: formalin-fixed paraffin-embedded
HNSCC: Head and neck squamous cell carcinoma
HPV: human papillomavirus
H&E: hematoxylin and eosin
ICIs: immune checkpoint inhibitors
LN: lymph node
PD-L1: programmed death-ligand 1
REP: rapid expansion protocol
R/M: Recurrent and/or metastatic
sTIL: stromal tumor-infiltrating lymphocytes
TCR: T-cell receptor
TIL: tumor-infiltrating lymphocytes
T_cm_: central memory T cells
T_eff_: effector T cells
T_em_: effector memory T cells
T_naive_: naïve T cells
