# Supplementary material for: Expansion of tumor-infiltrating lymphocytes from head and neck squamous cell carcinoma to assess the potential of adoptive cell therapy
Source: Cancer Immunol Immunother. 2024 Apr 17;73(6):101. doi: 10.1007/s00262-024-03691-9 (PMC11024072; doi:10.1007/s00262-024-03691-9)
Supplement: Supplementary file 1 — Supplementary Material 1 [file 262_2024_3691_MOESM1_ESM.docx]

Supplementary Table 1. Clinicopathologic characteristics of 21 HNSCC samples with available HPV results

|  | HPV negative | HPV positive |  |
| --- | --- | --- | --- |
|  | n=6 | n=15 | p value |
|  |  |  |  |
| Age | 63.0 ± 12.9 | 65.3 ± 5.7 | 0.694 |
|  |  |  |  |
| Sex |  |  |  |
| male | 6 (100.0) | 11 (73.3) | 0.281 |
| female | 0 (0.0) | 4 (26.7) |  |
|  |  |  |  |
| Sample type |  |  |  |
| LN | 3 (50.0) | 7 (46.7) | 1.000 |
| Tumor | 3 (50.0) | 8 (53.3) |  |
|  |  |  |  |
| AJCC T stage |  |  |  |
| 1 | 0 (0.0) | 1 (6.7) | 0.001 |
| 2 | 0 (0.0) | 12 (80.0) |  |
| 3 | 3 (50.0) | 2 (13.3) |  |
| 4 | 3 (50.0) | 0 (0.0) |  |
|  |  |  |  |
| AJCC N stage |  |  |  |
| 0 | 0 (0.0) | 1 (6.7) | 0.007 |
| 1 | 1 (16.7) | 12 (80.0) |  |
| 2 | 3 (50.0) | 2 (13.3) |  |
| 3 | 2 (33.3) | 0 (0.0) |  |
|  |  |  |  |
| Histology |  |  |  |
| WD | 0 (0.0) | 2 (13.3) | 1.000 |
| MD | 6 (100.0) | 13 (86.7) |  |
| PD | 0 (0.0) | 0 (0.0) |  |
|  |  |  |  |
| Location group |  |  |  |
| Oral cavity | 5 (83.3) | 0 (0.0) | <0.001 |
| Oropharynx | 1 (16.7) | 15 (100.0) |  |
| Larynx | 0 (0.0) | 0 (0.0) |  |
|  |  |  |  |
| Smoking history |  |  |  |
| (-) | 0 (0.0) | 5 (33.3) | 0.262 |
| (+) | 6 (100.0) | 10 (66.7) |  |
|  |  |  |  |
| Smoking duration (pack-years) |  |  |  |
| 0 | 0 (0.0) | 3 (33.3) | 0.433 |
| 1-20 | 3 (75.0) | 2 (22.2) |  |
| 21-40 | 0 (0.0) | 2 (22.2) |  |
| ≥41 | 1 (25.0) | 2 (22.2) |  |
|  |  |  |  |
| p16 immunoreactivity | |  |  |
| (-) | 6 (100.0) | 0 (0.0) | <0.001 |
| (+) | 0 (0.0) | 15 (100.0) |  |
|  |  |  |  |
|  |  |  |  |
| Stromal TIL  (%, mean ± SD) | 60.0 ± 15.8 | 59.3 ± 28.9 | 1.000 |
|  |  |  |  |
| PD-L1 CPS (22C3) | 45.0 ± 48.2 | 70.0 ± 43.6 | 0.825 |
|  |  |  |  |
| TILs per fragment  (x10^5, mean ± SD) | 1.6 ± 2.2 | 3.3 ± 5.1 | 0.841 |
|  |  |  |  |
| Total cultured TILs  (x10^6, mean ± SD) | 9.2 ± 13.7 | 17.2 ± 27.0 | 0.715 |
|  |  |  |  |

Supplementary Table 2. Clinicopathologic characteristics of 47 HNSCC samples between tumor and lymph node

|  | Lymph node | Tumor |  |
| --- | --- | --- | --- |
|  | n=17 | n=30 | p value |
|  |  |  |  |
| Age | 64.65 ± 12.19 | 61.00 ± 14.35 | 0.362 |
|  |  |  |  |
| Sex |  |  |  |
| male | 12 (70.6) | 23 (76.7) | 0.733 |
| female | 5 (29.4) | 7 (23.3) |  |
|  |  |  |  |
| AJCC T stage |  |  |  |
| 1 | 1 (5.9) | 1 (3.3) | 0.967 |
| 2 | 7 (41.2) | 11 (36.7) |  |
| 3 | 6 (35.3) | 11 (36.7) |  |
| 4 | 3 (17.6) | 7 (23.3) |  |
|  |  |  |  |
| AJCC N stage |  |  |  |
| 0 | 0 (0.0) | 9 (30.0) | 0.055 |
| 1 | 9 (52.9) | 10 (33.3) |  |
| 2 | 6 (35.3) | 7 (23.3) |  |
| 3 | 2 (11.8) | 4 (13.3) |  |
|  |  |  |  |
| Histology |  |  |  |
| WD | 3 (17.6) | 7 (23.3) | 0.833 |
| MD | 0 (0.0) | 1 (3.3) |  |
| PD | 14 (82.4) | 22 (73.3) |  |
|  |  |  |  |
| Location group |  |  |  |
| Oral cavity | 7 (41.2) | 20 (66.7) | 0.185 |
| Oropharynx | 9 (52.9) | 8 (26.7) |  |
| Larynx | 1 (5.9) | 2 (6.7) |  |
|  |  |  |  |
| Smoking history |  |  |  |
| (-) | 6 (35.3) | 14 (46.7) | 0.652 |
| (+) | 11 (64.7) | 16 (53.3) |  |
|  |  |  |  |
| Smoking duration (pack-years) |  |  |  |
| 0 | 1 (33.3) | 14 (46.7) | 0.687 |
| 1-20 | 2 (66.7) | 7 (23.3) |  |
| 21-40 | 0 (0.0) | 4 (13.3) |  |
| ≥41 | 0 (0.0) | 5 (16.7) |  |
|  |  |  |  |
| p16 immunoreactivity | |  |  |
| (-) | 3 (27.3) | 6 (42.9) | 0.677 |
| (+) | 8 (72.7) | 8 (57.1) |  |
|  |  |  |  |
| HPV ISH |  |  |  |
| (-) | 3 (30.0) | 3 (27.3) | 1.000 |
| (+) | 7 (70.0) | 8 (72.7) |  |
|  |  |  |  |
| TILs per fragment  (x10^5, mean ± SD) | 3.27 ± 4.57 | 1.31 ± 2.85 | 0.162 |
|  |  |  |  |
| Total cultured TILs  (x10^6, mean ± SD) | 24.39 ± 33.73 | 7.13 ± 19.41 | 0.095 |
|  |  |  |  |
| Stromal TIL  (%, mean ± SD) | 52.81 ± 31.20 | 45.67 ± 34.23 | 0.438 |
|  |  |  |  |
| PD-L1 CPS (22C3)* | 80.00 ± NA | 45.44 ± 37.55 | NA |
|  |  |  |  |

* PD-L1 test was performed in only one LN sample and comparative analysis was not available
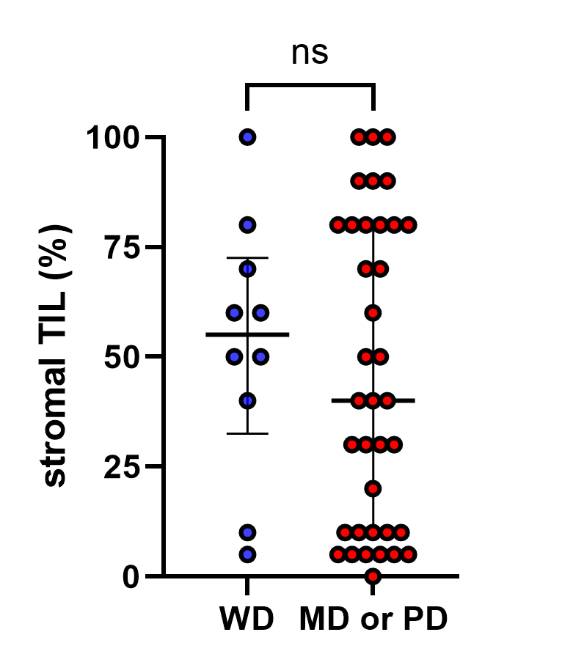


Supplementary Figure 1. Comparison of stromal tumor-infiltrating lymphocytes based on differentiation of squamous cell carcinoma

Supplementary Figure 2. Relationship between stromal tumor-infiltrating lymphocytes (sTIL), expanded TILs per fragment and total cultured TILs in tumor samples (upper panel) and lymph node samples (lower panel).


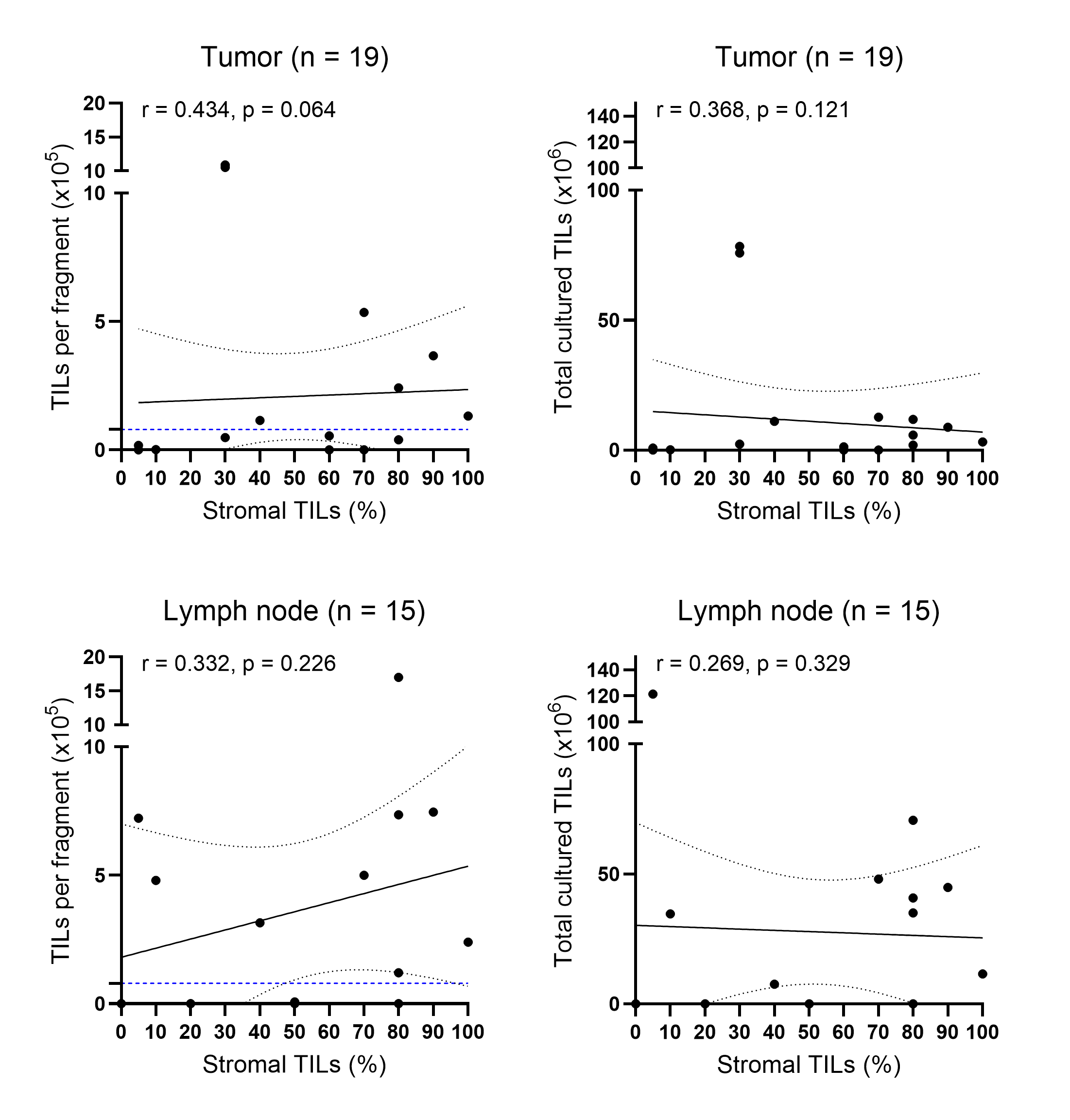

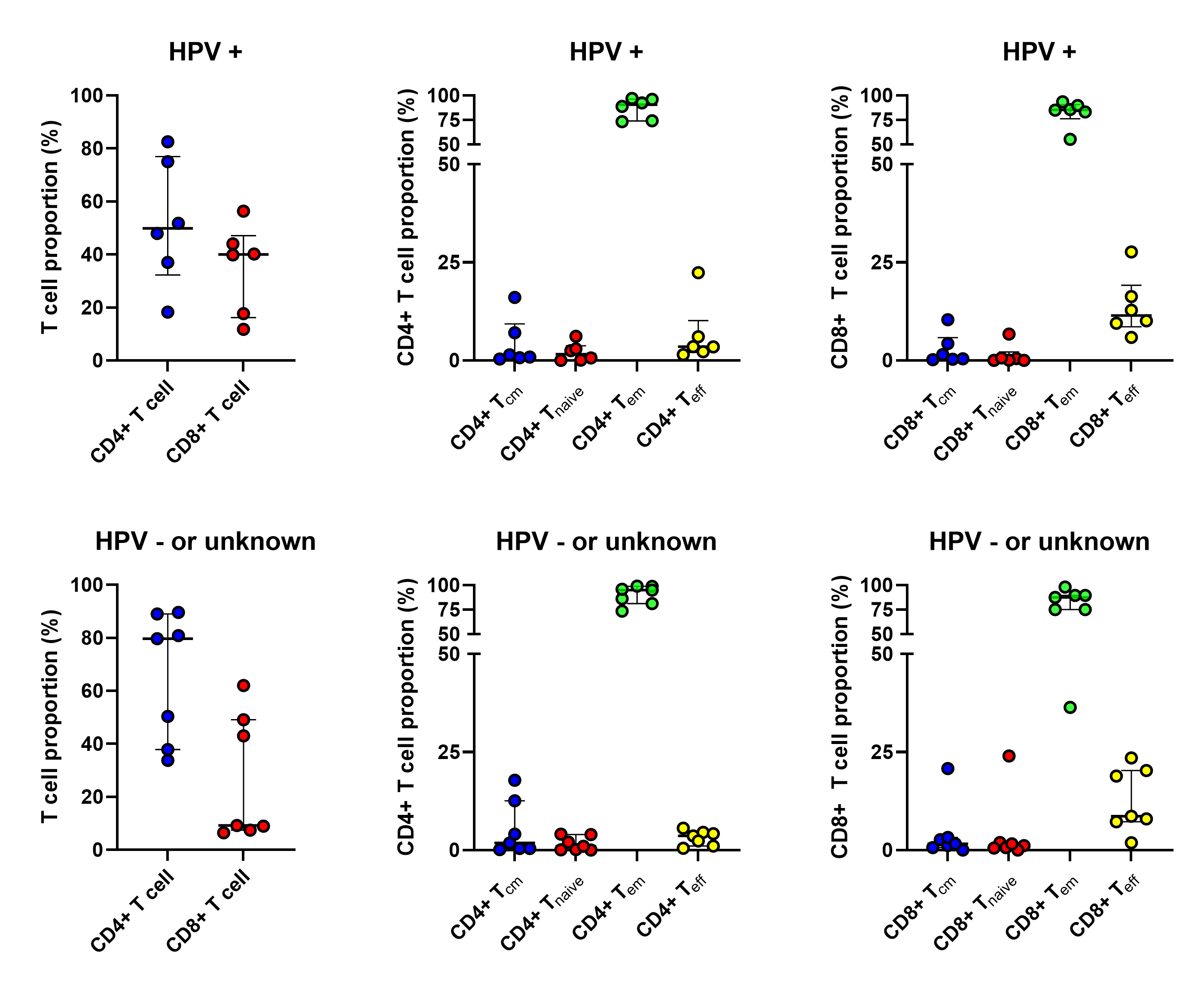


Supplementary Figure 3. TIL phenotypes of HPV-positive (n = 6) (upper panel) and HPV-negative/unknown (n = 7) samples (lower panel) with successful tumor-infiltrating lymphocytes expansion
